# The Association of Polycystic Ovarian Syndrome Among Reproductive-Aged Women With Consumption of Junk Food in Jeddah, Saudi Arabia

**DOI:** 10.7759/cureus.48299

**Published:** 2023-11-05

**Authors:** Ashraf Radwan, Abdulkreem A Al-Juhani, Abdulaziz A Alshehri, Ahmed A Alsumaili, Saeed K Aseri, Mohammed J Alzahrani, Dina M Qahwaji, Faysal Zaafarani

**Affiliations:** 1 Obstetrics and Gynecology, Faculty of Medicine, King Abdulaziz University, Jeddah, SAU; 2 Faculty of Medicine, King Abdulaziz University, Jeddah, SAU; 3 Clinical Nutrition, King Abdulaziz University, Jeddah, SAU

**Keywords:** follicle stimulating hormone, luteinizing hormone, facial acne, obesity, junk food consumption, polycystic ovary syndrome (pcos)

## Abstract

Objective: Polycystic ovarian syndrome (PCOS) is a common reproductive and endocrinological disorder that affects a significant percentage of women worldwide. Despite its high prevalence, the possible environmental factors that influence the development of PCOS, especially dietary habits, are still unclear. The aim of this study is to investigate the association between PCOS and junk food consumption among women of childbearing age in Jeddah, Saudi Arabia.

Methods: A cross-sectional study was conducted from February 2023 to April 2023 in 155 women diagnosed with PCOS according to the Rotterdam criteria. Participants aged 18-47 years and living in Jeddah, Saudi Arabia completed a validated 30-item electronic questionnaire that included sociodemographic information, medical history, PCOS symptoms, and fast food consumption patterns.

Results: The study revealed a high prevalence of obesity and overweight among PCOS patients, which was significantly associated with their diets high in carbohydrates, fats, and refined sugars. In addition, weight loss was difficult for these patients, which could be influenced by hormonal imbalances and dietary factors. In particular, fast food consumption showed a significant association with facial acne in PCOS patients, while some participants also experienced excessive hair growth on the face, abdomen, and back, which may be related to excessive fat intake.

Conclusion: This study provides evidence to support an association between junk food consumption and the prevalence of PCOS among women of reproductive age in Jeddah, Saudi Arabia. The findings suggest that dietary patterns may influence the development and treatment of PCOS, highlighting the need for lifestyle interventions as part of treatment strategies. Further studies with larger and more diverse populations, as well as longitudinal studies, are warranted to clarify causal relationships and gain a comprehensive understanding of the impact of dietary factors on the development and progression of PCOS. Identifying the potential effects of diet on PCOS can lead to more effective treatments and improve the overall health and well-being of those affected.

## Introduction

Nearly 6-10% of females suffer from the widespread reproductive and endocrinologic syndrome polycystic ovarian syndrome (PCOS) [1]. Infertility, insulin resistance, reduced glucose tolerance, and dyslipidemia can also be linked to the symptoms, which include an irregular menstrual cycle, polycystic ovaries, and hirsutism [2]. In 2010, the World Health Organization reported that PCOS affected over 116 million women worldwide. 3.4% of the world's female population is represented by this [3]. The number of people without a diagnosis is significant and may reach 75% even after consulting a doctor [4]. Women are frequently not diagnosed until they attempt conception [5]. In different nations and between endocrinologists and obstetrician-gynecologists, PCOS is diagnosed and treated differently. The NIH criteria are preferred by almost half of the endocrinologists and obstetricians-gynecologists in North America who employ the Rotterdam criteria. However, in Europe, about 75% of these medical professionals adhere to the Rotterdam standards. Furthermore, endocrinologists are more likely than obstetrician-gynecologists to use the Rotterdam diagnostic criteria [6]. Numerous research have shown a link between PCOS and gynecological malignancies [7]. Women with PCOS are three times more likely than typical women to develop endometrial cancer, according to a meta-analysis [8]. Why polycystic ovaries were only initially recognized in the early 1900s is an issue raised by the high contemporary frequency of polycystic ovaries in Western nations [9,10]. Environmental factors that contribute to PCOS susceptibility may overlap with those that contribute to obesity and metabolic disorders. Still, perfect unity between the two is unlikely, given that PCOS frequently precedes and can occur without being overweight or obese [11]. The challenge is to define a pathophysiological mechanism that accepts a contribution from both genetic and environmental determinants. There is also evidence that the development of a predisposition to PCOS begins during fetal development [12,13]. A prenatal or perinatal origin implies that environmental forces are at work [14], but it is uncertain what these influences are, as well as the genes and tissues they affect. Since then, numerous new industrial processes, nutritional components, food preservation and cooking techniques, and new environmental chemicals, poisons, and medications have developed and influenced human culture. Several of these have characteristics, biological actions, and exposure patterns that call for further research - all of these, alone or in combination, may be candidate causal agents. However, there are no limited previous similar studies conducted on the association between PCOS among reproductive-aged women and the consumption of junk food in the western region of Saudi Arabia. Moreover, the practices of causation correlation between junk food and PCOS are ineffective or missing. This study aims to determine the association between PCOS among reproductive-aged women and the consumption of junk food in the western region of Saudi Arabia.

## Materials and methods

This cross-sectional study was conducted in Jeddah, Saudi Arabia. It included 155 women diagnosed with PCOS to investigate the association between PCOS and the consumption of junk food from February 2023 to April 2023. The inclusion criteria consisted of women diagnosed with PCOS according to the Rotterdam criteria, which requires the presence of two out of three of the following criteria: hyperandrogenism (clinical or laboratory), chronic anovulation, and polycystic ovaries on ultrasound, aged between 18 and 47 years, and residing in Jeddah, Saudi Arabia, and able to speak Arabic or English. To ensure the accuracy of the study, several exclusion criteria were applied. Women who had not been diagnosed with PCOS were excluded from the study. Participants who had been previously diagnosed with hypothyroidism, Cushing's syndrome, steroid intake, hyperprolactinemia, or chronic renal insufficiency were also excluded. Additionally, individuals younger than 18 or older than 47 years, living outside of Jeddah, Saudi Arabia, or unable to speak Arabic or English were excluded from the study. Proper consent was obtained from the participants. Our team developed a 30-item electronic questionnaire under the supervision of a board-certified gynecology consultant and a clinical nutritionist. On the first page of the questionnaire, participants were informed about the background and objectives of the study. Participants were told they were free to withdraw at any time without giving a reason and that all information and opinions provided would be anonymous and confidential. The first eight items regarding socio-demographic and anthropometric characteristics included questions about age, residency, height (cm), weight (kg), and body mass index (BMI) calculated by dividing weight in kilograms by the square of height in meters (kg/m^2^), marital status, family income, parent's education level, and partner's education level. The five items regarding patient history included family history of PCOS, history of other OB/GYN diseases, history of diabetes mellitus and abnormal blood pressure, consumption of nicotine substances, and a stressful daily routine. The seven items regarding symptoms of PCOS included irregular periods (more or less than 28 days), facial acne, excessive hair growth on the face, stomach, and back, frontal hair loss, easy weight gain or trouble losing weight, occasions of abnormal blood sugar, and previous investigations for PCOS (ultrasound and blood test). The seven items regarding the fast food consumption pattern, which has been previously validated, included information about 12 food groups and beverages commonly consumed in Saudi Arabia. Each food group was judged using a Likert-like scale with four possible responses: never, more than once per month, more than once per week, and more than once per day. Additionally, participants reported the frequency, pattern, and portion size of fast food intake. Raosoft software was used to determine the appropriate sample size for this study, which was 384 at a confidence level of 95% and a margin of error of 5%. Participants were recruited from the King Abdulaziz University Hospital Jeddah, Saudi Arabia, clinical database from January 2013 to January 2023. The questionnaire data were collected directly through a telephone survey, wherein the participants were contacted via phone call and asked to respond to the questionnaire items.

Statistical analysis

Data analysis was done using RStudio (R version 4.2.2; R Foundation for Statistical Computing, Vienna, Austria). Descriptive statistics were used to express categorical variables (frequencies and percentages) and continuous variables (median and interquartile range [IQR]). A multiple-response analysis was used to analyze variables with multiple selections, including the beverages consumed with fast food and the time fast food is frequently consumed. A Fisher's exact test or a Pearson's chi-squared test was used to assess the association between PCOS symptoms and different patterns of fast food consumption. A p-value of <0.05 indicated statistical significance. Ethical approval was granted by the Biomedical Ethics Unit, Faculty of Medicine, KAU (HA-02-J-008). Informed verbal consent was obtained from all participants, which was compatible with our institutional approval. 

## Results

Initially, we received 209 responses on the online platform. We excluded 48 responses from individuals who did not reside in Jeddah and 29 responses from participants with a history of thyroid disease. Additionally, six participants were ineligible for the study due to their age range (<18 or >47 years). Therefore, the current study includes data from 146 eligible participants. Approximately half of the respondents aged 25 to <35 years (55.5%) were single (52.9%) and had no medical insurance that covers treatment (49.7%). 

Obesity and overweight were prevalent among 40.0% and 25.2%, respectively. Additionally, 4.5% of participants had a personal history of other OB/GYN diseases, and 24.8% of them had a chronic disease. About one-third of the participants had psychological stress, 1.9% had physical stress, and 28.6% had both physical and psychological stress. Focusing on the smoking history, only 7.1% of the respondents were active smokers and 8.5% used e-cigarettes. Furthermore, 13.7% of the respondents had ever used Hookah (shisha). More details about the history of smoking and other participants' characteristics are provided in Table 1.

**Table 1:** Demographic and clinical characteristics. PCOS, polycystic ovarian syndrome. *The variables have one missing value. ^¥^The variables have two missing values.

Parameter	Category	N (%)
Age	18 to <25	36 (23.2%)
	25 to <35	86 (55.5%)
	35 to 47	33 (21.3%)
Height (cm)	Median (IQR)	157.0 (154.0, 162.0)
Weight (kg)	Median (IQR)	69.0 (57.5, 81.0)
BMI (kg/m^2^)	Median (IQR)	27.5 (22.9, 32.7)
BMI category	Underweight	5 (3.2%)
	Healthy	49 (31.6%)
	Overweight	39 (25.2%)
	Obese	62 (40.0%)
Marital status	Single	82 (52.9%)
	Married	73 (47.1%)
Medical insurance covers the treatment	No	33 (21.3%)
	Yes	45 (29.0%)
	No medical insurance	77 (49.7%)
The educational level of parents*	Illiterate	25 (16.2%)
	High school or less	55 (35.7%)
	University or higher	74 (48.1%)
Family history of PCOS	No	62 (40.0%)
	Yes	48 (31.0%)
	Do not know	38 (24.5%)
Other OB/GYN history	Yes	7 (4.5%)
Personal history of chronic diseases	Yes	25 (24.8%)
Type of chronic diseases	Hypertension	10 (18.5%)
	Diabetes mellitus	15 (27.8%)
Cigarette smoking*	Non-smoker	143 (92.9%)
	Light smoker (1-10 C/D)	5 (3.2%)
	Moderate smoker (11-20 C/D)	4 (2.6%)
	Heavy smoker (more than 20 C/D)	2 (1.3%)
Use of e-cigarettes^¥^	No	140 (91.5%)
	60 ml per week	11 (7.2%)
	2 x 60 ml per week	2 (1.3%)
	3 x 60 ml per week	0 (0.0%)
Hookah (shisha) smoking^¥^	Never	132 (86.3%)
	Monthly	8 (5.2%)
	Weekly	8 (5.2%)
	Daily	5 (3.3%)
Having physical or psychological stress*	No	56 (36.4%)
	Psychological stress	51 (33.1%)
	Physical stress	3 (1.9%)
	Psychological and physical stress	44 (28.6%)

About half of the participants had a normal duration of the monthly period (60.4%), suffered from facial acne (53.5%), and had an unusual amount of blood in the menstrual cycle (44.4%). Considerable proportions of the respondents had frontal hair loss (66.5%), easily gain weight (67.7%) and hardly lose weight (61.7%). High blood pressure was prevalent among 8.4%, high glucose levels among 7.1%, and both high blood pressure and hyperglycemia among 3.2% (Table 2). 

**Table 2:** Characteristics of PCOS symptoms and their association with consuming fast food. PCOS, polycystic ovarian syndrome. ^*^The variables have one missing value. ^¥^The variables have two missing values.

Parameter	Category	Overall, N = 155	Eat restaurant fast food regularly			
			No, N = 28	Rarely, N = 52	Yes, N = 75	p-Value
Duration of the monthly period*	Normal range	93 (60.4%)	17 (18.3%)	31 (33.3%)	45 (48.4%)	0.749
	Less than 21 days	13 (8.4%)	4 (30.8%)	4 (30.8%)	5 (38.5%)	
	More than 35 days	48 (31.2%)	7 (14.6%)	16 (33.3%)	25 (52.1%)	
Amount of blood in your menstrual cycle^¥^	Low	40 (26.1%)	7 (17.5%)	14 (35.0%)	19 (47.5%)	0.625
	Heavy	45 (29.4%)	6 (13.3%)	13 (28.9%)	26 (57.8%)	
	Unusual	68 (44.4%)	14 (20.6%)	25 (36.8%)	29 (42.6%)	
Suffer from facial acne	No	72 (46.5%)	17 (23.6%)	29 (40.3%)	26 (36.1%)	0.016
	Yes	83 (53.5%)	11 (13.3%)	23 (27.7%)	49 (59.0%)	
Have excessive hair growth on the face, stomach, and back	No	66 (42.6%)	17 (25.8%)	22 (33.3%)	27 (40.9%)	0.078
	Yes	89 (57.4%)	11 (12.4%)	30 (33.7%)	48 (53.9%)	
Have frontal hair loss	No	52 (33.5%)	7 (13.5%)	16 (30.8%)	29 (55.8%)	0.372
	Yes	103 (66.5%)	21 (20.4%)	36 (35.0%)	46 (44.7%)	
Easy weight gain	No	50 (32.3%)	9 (18.0%)	16 (32.0%)	25 (50.0%)	0.955
	Yes	105 (67.7%)	19 (18.1%)	36 (34.3%)	50 (47.6%)	
Hard to lose weight*	No	59 (38.3%)	17 (28.8%)	23 (39.0%)	19 (32.2%)	0.001
	Yes	95 (61.7%)	10 (10.5%)	29 (30.5%)	56 (58.9%)	
Have occasions of the following:*	Never	125 (81.2%)	21 (16.8%)	43 (34.4%)	61 (48.8%)	0.520
	High glucose levels	11 (7.1%)	2 (18.2%)	3 (27.3%)	6 (54.5%)	
	High blood pressure	13 (8.4%)	2 (15.4%)	4 (30.8%)	7 (53.8%)	
	Both	5 (3.2%)	3 (60.0%)	1 (20.0%)	1 (20.0%)	

**Figure 1:**
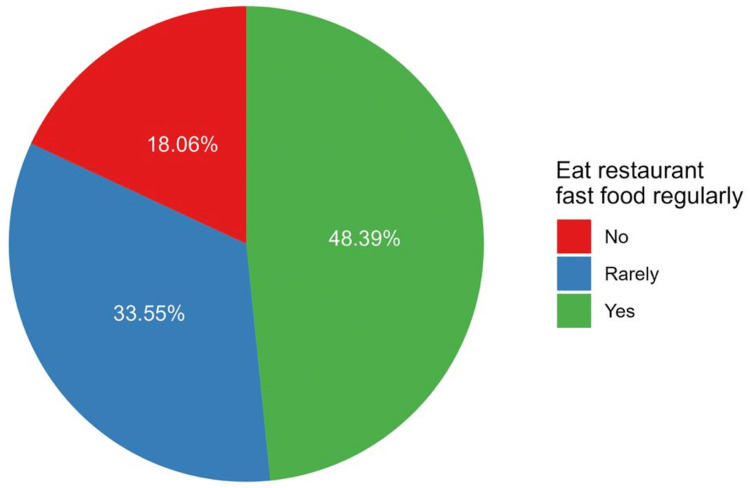
The percentages of patterns of fast food consumption among participants.

Out of the included participants, 75 respondents indicated that they consume restaurant fast food regularly, which represents 48.4% of the sample, whereas 33.6% of them rarely consumed fast food (Figure 1).

About one-third of the respondents declared that they consume fast food four times per week or less (30.3%) and consume fast food rich in carbohydrates four times per week or less (33.8%), fast food rich in fat four times per week or less (33.1%), and fast food rich in protein more than five times per week (32.5%). The majority of participants used to eat fast food in a normal portion size (61.3%, Table 3).

**Table 3:** Patterns of fast food consumption *The variables have one missing value. ^¥^The variable has five missing values.

Parameter	Category	N (%)
Frequency of consuming fast food in general	Once per week or less	95 (61.3%)
	4 times per week or less	47 (30.3%)
	More than 5 times per week	13 (8.4%)
Consumption of fast food rich in carbohydrates*	Never	3 (1.9%)
	Monthly or more	5 (3.2%)
	Once per week or less	37 (24.0%)
	4 times per week or less	52 (33.8%)
	More than 5 times per week	57 (37.0%)
Consumption of fast food rich in fat*	Never	7 (4.5%)
	Monthly or more	14 (9.1%)
	Once per week or less	49 (31.8%)
	4 times per week or less	51 (33.1%)
	More than 5 times per week	33 (21.4%)
Consumption of fast food rich in protein*	Never	1 (0.6%)
	Monthly or more	9 (5.8%)
	Once per week or less	48 (31.2%)
	4 times per week or less	46 (29.9%)
	More than 5 times per week	50 (32.5%)
Portion size of fast food that is usually eaten^¥^	Less than your needs	29 (19.3%)
	Normal	92 (61.3%)
	More than your needs	29 (19.3%)

**Figure 2:**
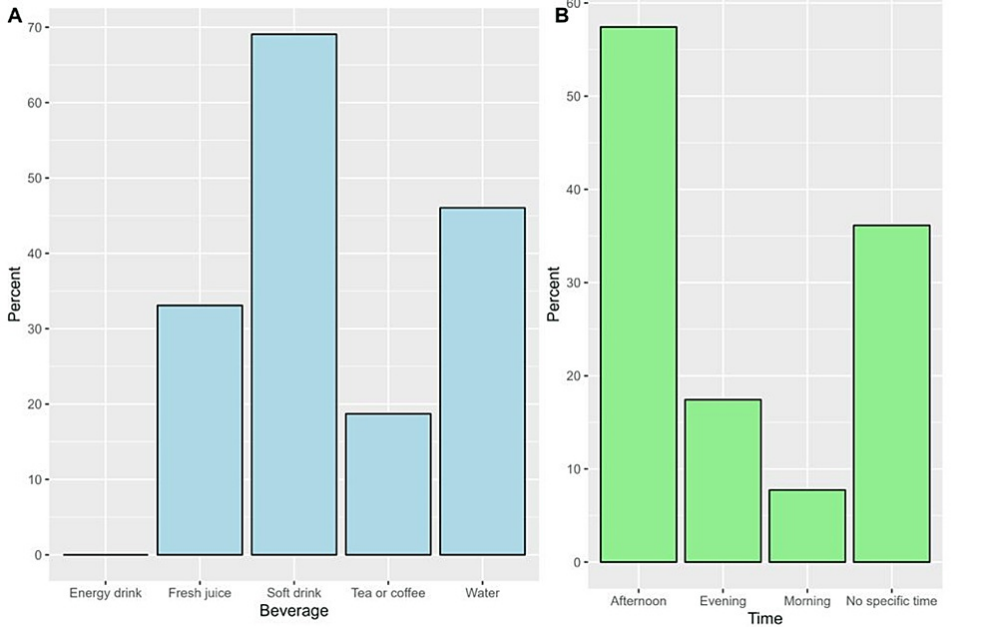
The proportions of beverages consumed with fast food (A) and the time periods at which participants used to consume fast food (B).

The majority of participants used to drink a beverage with fast food (90.8%). Among those who used to consume beverages, respondents consumed soft drinks more frequently (69.1%) and water (46.0%, Figure 2A). The most common time at which the respondents consumed fast food was in the afternoon (57.4%, Figure 2B). 

Factors associated with fast food consumption

Fast food consumption was significantly associated with having facial acne (59.0% vs 36.1% among those without facial acne, p = 0.016) and the inability to lose weight easily (58.9% vs 32.2%, p = 0.001). No significant associations were found between fast food consumption and other PCOS symptoms (Table 2).

Furthermore, there were no factors associated with the frequency of consuming fast food (Table 4).

**Table 4:** Factors associated with the frequency of consuming fast food.

Parameter	Category	Frequency of consuming fast food in general			
		Once per week or less, N = 95	4 times per week or less, N = 47	More than 5 times per week, N = 13	p-Value
Duration of the monthly period	Normal range	58 (62.4%)	30 (32.3%)	5 (5.4%)	0.461
	Less than 21 days	8 (61.5%)	3 (23.1%)	2 (15.4%)	
	More than 35 days	28 (58.3%)	14 (29.2%)	6 (12.5%)	
Amount of blood in your menstrual cycle	Low	25 (62.5%)	14 (35.0%)	1 (2.5%)	0.316
	Heavy	25 (55.6%)	13 (28.9%)	7 (15.6%)	
	Unusual	43 (63.2%)	20 (29.4%)	5 (7.4%)	
Suffer from facial acne	No	51 (70.8%)	15 (20.8%)	6 (8.3%)	0.050
	Yes	44 (53.0%)	32 (38.6%)	7 (8.4%)	
Have excessive hair growth on the face, stomach, and back	No	42 (63.6%)	18 (27.3%)	6 (9.1%)	0.770
	Yes	53 (59.6%)	29 (32.6%)	7 (7.9%)	
Have frontal hair loss	No	34 (65.4%)	13 (25.0%)	5 (9.6%)	0.583
	Yes	61 (59.2%)	34 (33.0%)	8 (7.8%)	
Easy weight gain	No	28 (56.0%)	15 (30.0%)	7 (14.0%)	0.233
	Yes	67 (63.8%)	32 (30.5%)	6 (5.7%)	
Hard to lose weight	No	42 (71.2%)	14 (23.7%)	3 (5.1%)	0.124
	Yes	52 (54.7%)	33 (34.7%)	10 (10.5%)	
Have occasions of the following	Never	73 (58.4%)	40 (32.0%)	12 (9.6%)	0.890
	High glucose levels	9 (81.8%)	2 (18.2%)	0 (0.0%)	
	High blood pressure	8 (61.5%)	4 (30.8%)	1 (7.7%)	
	Both	4 (80.0%)	1 (20.0%)	0 (0.0%)	

Regarding the portion size of fast food, a significantly higher proportion of participants who reported hard loss of weight used to consume more than the need (26.1% vs 8.8%, p = 0.002, Table 5).

**Table 5:** Factors associated with the portion size of fast food.

Parameter	Category	Portion size of fast food that is usually eaten			
		Less than your needs, N = 29	Normal, N = 92	More than your needs, N = 29	p-Value
Duration of the monthly period	Normal range	17 (18.5%)	59 (64.1%)	16 (17.4%)	0.853
	Less than 21 days	3 (25.0%)	6 (50.0%)	3 (25.0%)	
	More than 35 days	9 (20.0%)	27 (60.0%)	9 (20.0%)	
Amount of blood in your menstrual cycle	Low	10 (25.6%)	20 (51.3%)	9 (23.1%)	0.702
	Heavy	8 (18.6%)	27 (62.8%)	8 (18.6%)	
	Unusual	11 (16.7%)	43 (65.2%)	12 (18.2%)	
Suffer from facial acne	No	11 (16.4%)	47 (70.1%)	9 (13.4%)	0.120
	Yes	18 (21.7%)	45 (54.2%)	20 (24.1%)	
Have excessive hair growth on the face, stomach, and back	No	12 (18.8%)	44 (68.8%)	8 (12.5%)	0.156
	Yes	17 (19.8%)	48 (55.8%)	21 (24.4%)	
Have frontal hair loss	No	5 (10.0%)	35 (70.0%)	10 (20.0%)	0.116
	Yes	24 (24.0%)	57 (57.0%)	19 (19.0%)	
Easy weight gain	No	13 (26.5%)	29 (59.2%)	7 (14.3%)	0.227
	Yes	16 (15.8%)	63 (62.4%)	22 (21.8%)	
Hard to lose weight	No	18 (31.6%)	34 (59.6%)	5 (8.8%)	0.002
	Yes	11 (12.0%)	57 (62.0%)	24 (26.1%)	
Have occasions of the following	Never	24 (19.5%)	78 (63.4%)	21 (17.1%)	0.080
	High glucose levels	3 (27.3%)	4 (36.4%)	4 (36.4%)	
	High blood pressure	0 (0.0%)	8 (66.7%)	4 (33.3%)	
	Both	2 (50.0%)	2 (50.0%)	0 (0.0%)	

## Discussion

Our study unequivocally confirmed that there is a high prevalence of obesity and being overweight among patients with PCOS. We strongly believe that this is directly linked to their dietary habits. Our findings revealed that these patients consume a diet that is rich in carbohydrates, fats, and refined sugars, which makes weight loss a challenging task for them. We are confident that this is the root cause of the problem, and our findings align with a systematic review and meta-analysis conducted by Romitti et al. that demonstrated that PCOS patients from different populations are significantly more likely to have autoimmune thyroid disease compared to controls [15].

Furthermore, we observed a noteworthy correlation between the consumption of fast food and the presence of facial acne in PCOS patients. Kim et al.'s study found that BMI affects menstrual irregularity, facial acne, and hirsutism in PCOS women [16]. Additionally, excessive hair growth on the face, abdomen, and back was observed in some patients, which we speculate could be related to the intake of excessive fats. This is thought to occur because these fats may be converted to androgen in peripheral tissues, a key factor in hair growth. However, we did not find any significant association between fast food consumption and other signs and symptoms of PCOS in our study. A study by Basu et al. indicates that PCOS patients have elevated levels of serum amyloid A activity and salivary cortisol compared to age-matched controls. This suggests that women affected by PCOS may experience an exaggerated response from the central stress systems. The findings imply a potential link between stress and PCOS, as the increased stress-related markers may disrupt hormonal balance and influence the condition's development or severity [17], which supports our finding that about one-third of PCOS patients had psychological stress.

In future studies, it is essential to increase the sample size and include diverse populations to ensure the findings can be generalized. A longitudinal cohort study with rigorous follow-up is recommended to establish a causal relationship between fast food consumption and patient's PCOS. The investigation should also focus on hormonal analysis, particularly examining follicle-stimulating hormone (FSH) and luteinizing hormone (LH) responses to fast food intake and BMI, to understand their impact on PCOS better. Additionally, researchers should carefully consider confounding factors, such as medication usage and exercise habits, in their study design. Including control groups and accurate dietary assessment tools will further support the significance of any associations found. Moreover, lifestyle factors like stress, sleep patterns, and physical activity should be considered during the analysis to provide a comprehensive understanding of the relationship between diet and PCOS. 

The present cross-sectional study aimed to explore the association between PCOS among reproductive-aged women and the consumption of junk food in the western region of Saudi Arabia. Our findings suggest a significant relationship between the intake of junk food and the prevalence of PCOS in the target population. This association can be attributed to several factors, including hormonal imbalances, insulin resistance, drug intake, and inflammation, which are known contributors to the development of PCOS. This finding is consistent with Hajivandi et al.'s study finding in which adolescent girls with PCOS consume a lot of unhealthy foods such as fast food, soft drinks, sweets, and junk food [18].

The main strength of our study is that this is the first cross-sectional research to examine the association between PCOS and the consumption of junk food among reproductive-aged women in Jeddah city in Saudi Arabia. Also, the questionnaire is validated for fast food consumption, including a comprehensive set of questions covering demographic and clinical characteristics, PCOS symptoms, and fast food consumption patterns, which provides a detailed understanding of the study population and the variables of interest. In addition, the questionnaire was administered verbally to participants who had already been diagnosed with PCOS from the KAUH database. The present study also has limitations. The current study is of cross-sectional design, which restricts our ability to make causal inferences. Longitudinal studies are needed to establish a temporal relationship between junk food consumption, the development of PCOS, and the worsening of its symptoms. One of the limitations of our study is that the participants were only involved from a KAUH hospital setting and had a low sample size. This may introduce a selection bias as it does not capture the full diversity of reproductive-aged women with PCOS in the broader population of Jeddah city in Saudi Arabia. The findings may not be generalizable to PCOS patients who do not seek care at hospitals or those residing in other settings, such as primary healthcare centers or private clinics. The study relies on a phone survey, which may be subject to recall bias and social desirability bias. 

## Conclusions

To sum up, our study found a high prevalence of obesity and overweight in PCOS patients linked to their consumption of a diet rich in carbohydrates, fats, and refined sugars. Weight loss was challenging, likely due to hormonal imbalances and dietary factors. We observed a significant correlation between fast food consumption and facial acne in PCOS patients. Future research should include more extensive and diverse populations, and longitudinal studies are needed to establish causal relationships. Understanding dietary influences on PCOS can improve management and treatment strategies for affected individuals.
